# Effectiveness and safety of first-generation protease inhibitors in real-world patients with hepatitis C virus genotype 1 infection in Brazil: a multicenter study

**DOI:** 10.6061/clinics/2017(06)08

**Published:** 2017-06

**Authors:** Luciana Azevedo Callefi, Cristiane Alves Villela-Nogueira, Simone de Barros Tenore, Dimas Carnaúba-Júnior, Henrique Sérgio Moraes Coelho, Paulo de Tarso A. Pinto, Letícia Cancella Nabuco, Mário Guimarães Pessoa, Maria Lucia Cardoso Gomes Ferraz, Paulo Roberto Abrão Ferreira, Ana de Lourdes Candolo Martinelli, Silvana Gama Florencio Chachá, Adalgisa de Souza Paiva Ferreira, Alessandra Porto de Macedo Bisio, Carlos Eduardo Brandão-Mello, Mário Reis Álvares-Da-Silva, Tânia Reuter, Claudia Alexandra Pontes Ivantes, Renata de Mello Perez, Maria Cássia Jacintho Mendes-Correa

**Affiliations:** I Departamento de Molestias Infecciosas e Parasitarias, Faculdade de Medicina, Universidade de Sao Paulo (USP), SP, BR; II Servico de Hepatologia, Departamento de Clinica Medica, Universidade Federal do Rio de Janeiro (UFRJ), RJ, BR; III Centro de Referencia e Treinamento em DST/Aids, SP, BR; IV Centro de Doencas Hepaticas (CDH), RJ, BR; V Setor de Gastro/Hepatologia, Hospital Federal dos Servidores do Estado do Rio de Janeiro (HFSE), RJ, BR; VI Departamento de Gastroenterologia e Hepatologia, Faculdade de Medicina, Universidade de Sao Paulo (USP), SP, BR; VII Disciplina de Gastroenterologia, Escola Paulista de Medicina (EPM), Universidade Federal de Sao Paulo (UNIFESP), SP, BR; VIII Disciplina de Infectologia, Escola Paulista de Medicina (EPM), Universidade Federal de Sao Paulo (UNIFESP), SP, BR; IX Divisao de Gastroenterologia, Departamento de Clinica Medica, Faculdade de Medicina de Ribeirao Preto (FMRP), Universidade de Sao Paulo (USP), SP, BR; X Centro de Pesquisa Clinica, Hospital Universitario Presidente Dutra (HUPD), Universidade Federal do Maranhao (UFMA), MA, BR; XI Disciplina de Clinica Medica e Gastroenterologia, Universidade Federal do Estado do Rio de Janeiro (UNIRIO), RJ, BR; XII Departamento de Medicina Interna, Universidade Federal do Rio Grande do Sul (UFRGS), RS, BR; XIII Ambulatorio HIV/AIDS/ Hepatites Virais, Universidade Federal do Espirito Santo (UFES), ES, BR; XIV Centro de Orientacao e Aconselhamento, Secretaria Municipal de Saude (SMS), PR, BR; XV Servico de Gastroenterologia, Universidade do Estado do Rio de Janeiro (UERJ), RJ, BR; XVI Laboratorio de Virologia-LIM 52, Instituto de Medicina Tropical (IMT), SP, BR

**Keywords:** Protease inhibitors, Safety, Hepatitis C, Chronic, Therapeutics

## Abstract

**OBJECTIVE:**

To evaluate the effectiveness and safety of first-generation protease inhibitors for the treatment of genotype 1 hepatitis C virus-infected patients at Brazilian reference centers.

**METHODS:**

This multicenter cross-sectional study included hepatitis C virus genotype 1 monoinfected patients treated with Peg-interferon, ribavirin, and either boceprevir (n=158) or telaprevir (n=557) between July 2013 and April 2014 at 15 reference centers in Brazil. Demographic, clinical, virological, and adverse events data were collected during treatment and follow-up.

**RESULTS:**

Of the 715 patients, 59% had cirrhosis and 67.1% were treatment-experienced. Based on intention-to-treat analysis, the overall sustained viral response was 56.6%, with similar effectiveness in both groups (51.9% for boceprevir and 58% for telaprevir, *p*=0.190). Serious adverse events occurred in 44.2% of patients, and six deaths (0.8%) were recorded. Cirrhotic patients had lower sustained viral response rates than non-cirrhotic patients (46.9% *vs.* 70.6%, *p*<0.001) and a higher incidence of serious adverse events (50.7% *vs.* 34.8%, *p*<0.001). Multivariate analysis revealed that sustained viral response was associated with the absence of cirrhosis, viral recurrence after previous treatment, pretreatment platelet count greater than 100,000/mm^3^, and achievement of a rapid viral response. Female gender, age>65 years, diagnosis of cirrhosis, and abnormal hemoglobin levels/platelet counts prior to treatment were associated with serious adverse events.

**CONCLUSION:**

Although serious adverse events rates were higher in this infected population, sustained viral response rates were similar to those reported for other patient cohorts.

## INTRODUCTION

Infection with hepatitis C virus (HCV) is one of the leading causes of chronic liver disease worldwide 1. The World Health Organization 2 currently estimates that 110 million people have a history of HCV infection, and of these, 80 million are chronically infected with this virus 3. Until 2011, the standard treatment for HCV consisted of the use of PEGylated interferon (Peg-IFN) and ribavirin (RBV), which achieved a sustained viral response (SVR) rate of approximately 50% in patients with HCV genotype 1 4,5. Unfortunately, this regimen involved the development of significant adverse events (SAEs) and a high rate of treatment discontinuation 2. In 2011, the first direct-acting antiviral agents were introduced into clinical practice for the treatment of chronic hepatitis C 6-9. The protease inhibitors boceprevir (BOC) and telaprevir (TVR) were the first direct-acting antiviral agents used, and these drugs required co-administration with Peg-IFN and RBV, together constituting the so-called triple therapy. Triple therapy improved the response rates of genotype 1 patients in clinical studies and achieved SVR rates of 66–75% in treatment-naïve patients 6,7, 75–88% in relapsing patients (relapsers), 52–59% in partial responders, and 29–33% in null responders 8,9.

In addition to protease inhibitors, other classes of direct-acting antiviral agents have gradually been introduced into clinical practice, including NS5A and NS5B inhibitors 10,11. In this new therapeutic era, the combination of different classes of drugs has significantly increased the rate of therapeutic success for the treatment of chronic hepatitis C 10,11. Moreover, these new combinations are generally safer and do not require the use of interferon. Given its benefits, this new therapeutic modality is currently the therapy of choice for the treatment of chronic hepatitis C worldwide 10,11.

Between 2013 and 2015 in Brazil, triple therapy with BOC and TVR was the standard treatment for patients infected with HCV genotype 1 12-13. The objective of this study was to evaluate the effectiveness and safety of this therapeutic modality in patients with chronic hepatitis C genotype 1 who were treated in 15 different reference centers in Brazil. Additionally, the predictive factors for the achievement of SVR and for the occurrence of SAEs associated with this therapy were evaluated.

## PATIENTS AND METHODS

### Patients

This multicenter cross-sectional study included patients chronically monoinfected with HCV genotype 1 (treatment-naïve or previously treated with Peg-IFN and RBV) who were treated with Peg-IFN (α2a or α2b), RBV and either BOC or TVR according to the guidelines of the Brazilian Ministry of Health 12,13 in 15 reference centers in Brazil. The guidelines of the Brazilian Ministry of Health prioritized triple therapy for patients monoinfected with HCV genotype 1 with advanced liver disease (Metavir F3 or F4) or evidence of portal hypertension and compensated hepatic cirrhosis (Child-Pugh≤6), previously treated patients with grade 2 fibrosis (Metavir F2), or patients with extrahepatic manifestation 12,13.

All patients who started treatment in these centers between July 2013 and April 2014 were eligible for this study. The exclusion criteria were individuals younger than 18 years of age and individuals co-infected with human immunodeficiency virus.

This study was approved by the Ethics Committee for the Analysis of Research Projects (Comissão de Ética para Análise de Projetos de Pesquisa–CAPPesq) of the Clinics Hospital of the Medical School of the University of São Paulo (Hospital das Clínicas da Faculdade de Medicina da Universidade de São Paulo – HC-FMUSP) under protocol no. 11,934.

## METHODS

### Data collection and variables analyzed

Data were collected using a standardized questionnaire. The variables selected for analysis were grouped into the following different categories: 1) variables related to the patient: age, gender, body mass index, and comorbidities; 2) variables related to HCV infection before the present treatment: genotype 1 subtype, presence of extrahepatic manifestations, presence of previous hepatic decompensation, the last value recorded in medical records for hemoglobin levels, platelet counts, albumin levels, HCV viral load, and staging of liver fibrosis; and 3) variables related to the treatment of hepatitis C: history of previous treatment as well as the type of viral response observed and the occurrence of SAEs during the current treatment. The variables analyzed in our study were standardized according to specific considerations. For the laboratory test conducted prior to treatment, the following results were considered abnormal: 1- hemoglobin levels <12 g/dL for female patients and <13 g/dL for male patients; 2- platelet count <100,000 per mm^3^; and 3- albumin levels <3.5 g/dL. Regarding hepatic fibrosis staging, liver biopsy results obtained using the Metavir Cooperative Study Group 14 classification and/or liver elastography were considered. The corresponding cut-off values of transient elastography for Metavir were 7.1 to 9.4 kPa = F2, 9.5 to 12.4 kPa = F3, and ≥12.5 kPa = F4 15. Patients were also classified as F4 if they had clinical characteristics of liver cirrhosis defined by the presence of portal hypertension. There was no histological or elastographic evaluation for this group.

Regarding the type of response observed after previous treatment with Peg-IFN and RBV, non-responders were considered those patients with any of the following types of viral responses as a result of previous treatments: partial responders - reduction of at least 2 log_10_ in HCV RNA levels at week 12 of treatment but with detectable HCV RNA at week 24; and null responders - reduction under 2 log_10_ in HCV RNA levels at week 12 of treatment. In addition, those who did not have a defined viral response to previous treatment were considered non-responders.

SAEs were defined as events that resulted in death, represented a threat to life, required hospitalization or prolongation of an existing hospitalization, resulted in persistent or significant disability, or promoted congenital malformation or anomaly, as defined in previous studies 16. We also followed previously established guidelines 17,18 with respect to the presence of rash grade 3 or 4 or the presence of laboratory adverse events grade 3 or 4.

### Treatment

The decision to initiate treatment and the choice between BOC or TVR and Peg-IFN-α2a (180 mg) or Peg-IFN-α2b (1.5 μg/kg) was entirely at the discretion of the attending physician of each participating service, according to the guidelines of the Brazilian Ministry of Health and the product manufacturers 19,20.

### Monitoring of HCV RNA

HCV RNA levels were measured at weeks 4 and 12 of treatment, at the end of therapy and at weeks 12 and/or 24 after the end of therapy. We performed real-time polymerase chain reaction (PCR) with a detection limit of 12 IU/mL (Abbott Molecular, Des Plaines, IL) 21 for these measurements.

The rapid viral response (RVR) was defined as undetectable HCV RNA (<12 IU/mL) at week 8 for those treated with BOC and week 4 for those treated with TVR (we considered week 8 to be when “lead in” was performed).

### Evaluation of effectiveness

Effectiveness was evaluated using the intention-to-treat principle. The primary outcome of the study was SVR, which was defined as undetectable levels of HCV RNA (<12 IU/mL) at least 12 weeks after discontinuation of treatment.

### Assessment of safety

The safety of triple therapy using BOC and TVR was assessed by determining the occurrence of SAEs during treatment.

### Statistical analysis

A descriptive analysis of the baseline demographic and clinical characteristics was performed. The results are presented as frequency tables for qualitative variables; central tendency and dispersion measures were estimated for quantitative variables. Pearson’s chi-square test was used to compare the patients according to the treatment provided. Fisher’s exact test was used for situations in which this test was not appropriate. Student’s *t*-test or the Mann-Whitney U-test was applied for continuous variables as required.

The prevalence of treatment effectiveness, rate of SAE occurrence, and respective 95% confidence intervals (95% CIs) were calculated. The factors associated with the effectiveness of treatment and SAE occurrence were determined in patients with chronic hepatitis C by performing a bivariate analysis and determining the prevalence ratios (PRs) and respective 95% CIs.

Variables with *p*-values smaller than 0.20 as determined by performing bivariate analysis were selected for multivariate analysis. The Poisson regression model with robust variance was used in the bivariate and multivariable analyses. PRs and their 95% CIs were estimated for each of the variables in each final model. A significance level of 5% was adopted.

## RESULTS

### Patient characteristics

A total of 715 adult patients with chronic HCV infection were included in this study. The majority of patients were male (56.1%), with a mean age of 54.1±10.1 years; 267 individuals (37.3%) had HCV subtype 1a, and 315 (44.1%) had subtype 1b. Among the studied patients, 422 (59%) had liver cirrhosis, and 67.1% were treatment-experienced. In total, 557 patients (77.9%) received treatment with Peg-IFN, RBV, and TVR, and 158 (22.1%) patients received treatment with Peg-IFN, RBV, and BOC.

Table 1 shows the baseline characteristics of the patients according to treatment group (BOC *vs*. TVR). Patients treated with BOC reported a higher prevalence of extrahepatic manifestations before treatment and a more frequent history of previous hepatic decompensation than those treated with TVR (13.3 *vs.* 3.9%, *p*<0.001 and 6.3 *vs.* 2.2%, *p*=0.007, respectively). However, there was a predominance of treatment-naïve patients in the group treated with TVR compared to the number in the group treated with BOC (35.2 *vs.* 24.7%, *p*=0.045). Regarding the laboratory tests conducted before treatment, abnormal hemoglobin levels [<12 g/dL (women), <13 g/dL (men)] and platelet counts <100,000/mm^3^ in the BOC group were significantly more frequent in the BOC group than in the TVR group (10.9 *vs.* 4.6%, *p*=0.004 and 24.4 *vs.* 17.1%, *p*=0.040).

### Effectiveness

**Figure 1:**
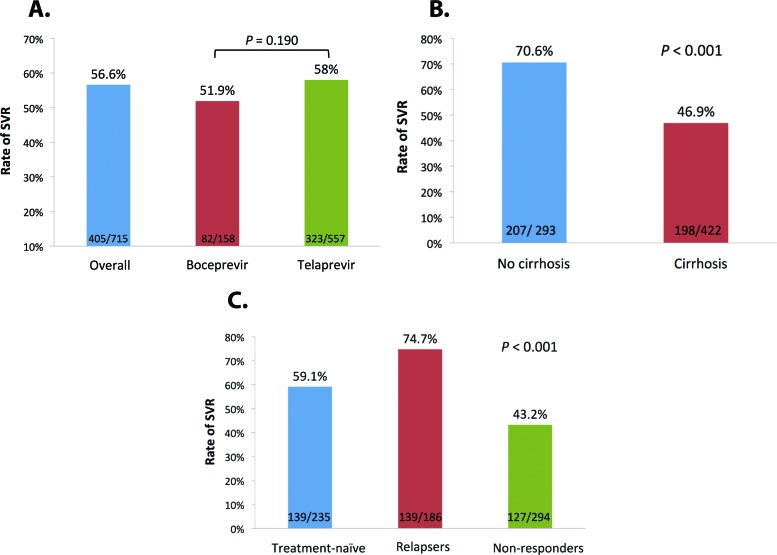
Rates of SVR in patients with chronic hepatitis C genotype 1 treated with first-generation protease inhibitors. (A) Overall SVR according to the protease inhibitor used. (B) SVR according to the presence of cirrhosis. (C) SVR according to the history of previous treatment.

According to the intention-to-treat analysis (n=715), the overall percentage of patients with SVR was 56.6% (95% CI, 52.9–60.3%). The effectiveness of treatment between the groups that received BOC or TVR was similar (51.9 *vs.* 58.0%; *p*=0.190). However, the SVR rate was higher in patients without cirrhosis than in patients with cirrhosis (70.6 *vs.* 46.9%, *p*<0.001). Taking into account a history of previous treatment, SVR rates were 59.1% for treatment-naïve patients, 74.3% for relapsers, and 43.2% for non-responders (60% for prior partial responders and 33.5% for null responders) (*p*<0.001) (Figure 1). Of the 310 patients who did not achieve SVR, 148 (47.7%) patients had viral failure during treatment, 87 (28.1%) patients discontinued treatment because of adverse events, 69 (22.3%) patients relapsed, and 11 (3.5%) patients abandoned treatment.

The results obtained for the 2 groups of patients were then combined to identify factors associated with treatment effectiveness. The results of the univariate and multivariate analyses are shown in Table 2. The variables defined as predictors of achievement of SVR were the presence of liver cirrhosis (PR, 0.77; 95% CI, 0.68–0.87; *p*<0.001), history of previous treatment in non-responders (PR, 0.69; 95% CI, 0.59–0.81; *p*<0.001), being treatment-naïve (PR, 0.83; 95% CI, 0.73–0.95; *p*<0.001) *vs.* being a relapser, a platelet count <100,000/mm^3^ prior to treatment (PR, 0.6; 95% CI, 0.46–0.77; *p*<0.001), and the presence of RVR (PR 1.9; 95% CI, 1.58–2.30; *p*<0.001).

### Safety

Of the 715 patients, 316 (44.2%; 95% CI, 40.5–47.9) had at least one SAE episode. A total of 581 SAE episodes were observed. The SAE rate was significantly higher among patients with cirrhosis than among patients without cirrhosis (50.7 *vs.* 34.8%, *p*<0.001). Premature discontinuation of treatment in cases of SAE was observed in 121 (16.9%) patients, and the most common SAEs were hepatic decompensation (n=28), anemia (n=23), and infection (n=20). Furthermore, six deaths (0.8%) were observed among the treated patients. Most of these cases were associated with infections, including spontaneous bacterial peritonitis (n=1), septic arthritis (n=1), and skin sepsis (n=1). Other causes of death included hepatorenal syndrome (n=1), pulmonary embolism (n=1), and cerebral aneurysm (n=1).

Table 3 compares patient safety profiles according to treatment group (BOC *vs.* TVR). Patients treated with TVR had a higher frequency of degree 3 or 4 anemia than patients treated with BOC and an increased need for blood transfusion (26.9 *vs.* 19.0%, *p*=0.042 and 13.5 *vs.* 6.3%, *p*=0.014, respectively).

According to a multivariate analysis, the factors associated with the occurrence of SAEs were female gender (PR, 1.42; 95% CI, 1.21–1.67; *p*<0.001), age >65 years (PR, 1.32; 95% CI 1.07–1.62; *p*=0.008), the presence of liver cirrhosis (PR, 1.25; 95% CI, 1.04–1.52; *p*=0.019), and abnormal hemoglobin levels or platelet counts before treatment (PR, 1.56; 95% CI, 1.23–1.98; *p*<0.001 and PR, 1.54; 95% CI, 1.30–1.82; *p*<0.001, respectively) (Table 4).

## DISCUSSION

Based on our results, 56.6% of patients with chronic hepatitis C treated with therapies involving the use of BOC or TVR achieved SVR. Multivariate analysis indicated that the factors associated with the achievement of SVR were the absence of cirrhosis, a history of relapse after previous treatment with Peg-IFN and RBV, a platelet count >100,000/mm^3^ before therapy, and the presence of RVR. Regarding treatment safety, approximately 44.2% of study patients had SAEs. Multivariate analysis indicated that the factors associated with the occurrence of SAEs were the presence of liver cirrhosis, female gender, age >65 years, and abnormal hemoglobin levels or platelet counts before treatment.

Comparison of our results with those observed in other large real-life cohorts involving the use of triple therapy with BOC or TVR suggested the rate of SVR was similar to that observed in previous studies, in which this rate ranged between 44% and 58% 22-24 (Table 5). However, notably, in our sample there was a higher frequency of patients with cirrhosis (59%) than in the samples of other real-life studies, in which this rate ranged between 16% and 44% 22-24. To a certain extent, these results suggested a slightly higher SVR rate in our study considering our sample had a higher frequency of patients with advanced disease. In addition, our results constitute a profile of patients primarily treated in Brazil, i.e., patients with advanced liver disease, which is in accordance with the guidelines recommended by the Ministry of Health of Brazil.

The presence of liver cirrhosis has often been associated with a lower likelihood of SVR in the treatment of chronic hepatitis C, regardless of the treatment used 23-26. The mechanisms that determine a lower likelihood of cure are poorly understood. However, these factors likely include impaired immune response in patients with cirrhosis and lower responsiveness to the proposed treatment, limited distribution of drugs in the compromised liver parenchyma, and factors associated with drug toxicity in this group of patients 27. Our results corroborate this hypothesis, as we observed an SVR rate of 46.9% in patients with cirrhosis and 70.6% in patients without cirrhosis.

With respect to the SVR rates observed in our study for non-responders to previous treatments with Peg-IFN and RBV, our results corroborate those obtained in registration studies with BOC 6,8 and TVR 7,9 and in studies with real-life cohorts 23,28-30. Overall, relapsers have a higher likelihood of achieving SVR after triple therapy with BOC or TVR. In phase 3 studies with these drugs, relapsers were the most eligible candidates for triple therapy with BOC and TVR and achieved SVR rates between 75% 8 and 88% 9, respectively. Our study corroborates this finding, given the SVR rate of 74.7% observed among relapsers to previous treatments with Peg-IFN and RBV. Among non-responders, this rate was 43.2% in our sample, which is similar to that reported in clinical studies using BOC and TVR (29-59%, including partial responders and null responders) 8,9.

RVR was the best predictor of treatment success (SVR) in our cohort (OR 1.9, 95% CI, 1.58–2.30) and in other clinical studies 30,31. However, in our study, the impact of extended RVR was not assessed because this information was not available for analysis in 21% of patients.

Regarding treatment safety, we observed a high prevalence of SAEs in our study group (44.2%); this rate was higher than that reported in phase 3 studies for BOC 8 and TVR 7,9 and in other real-life studies 22,28,32,33 (Table 5). However, this result was similar to that obtained in the CUPIC study (49.9%) 29, likely due to the large number of patients with advanced liver disease in our study, which was conducted at tertiary referral centers where patients usually have diseases that are more complex. Based on our results, SAEs occurred in 50.7% of patients with cirrhosis and 34.8% of patients without cirrhosis. The higher prevalence of SAEs in cirrhotic patients is also in accordance with other previously published studies 29,32.

The most important factors associated with the occurrence of SAEs were the presence of liver cirrhosis, female gender, age >65 years, and abnormal hemoglobin levels or platelet counts before treatment. All of these factors have been extensively described in studies similar to ours 29,32. These findings underscore the strong association between the occurrence of SAEs during the treatment of hepatitis C and the presence of advanced liver disease 29,32.

Despite the high prevalence of SAEs in our study, the rate of discontinuation of therapy due to adverse events was low (16.9%) compared to rates observed in real-life studies 29,33,34 and was similar to that reported for the REALIZE registration study 9. These rates vary widely in the literature, ranging between 6.8% and 21.1% 22,28,29,33,34. This wide variability may reflect the clinical heterogeneity of the groups of patients included in the different studies. The low rate of treatment discontinuation observed in our study may be justified by the timeframe for the introduction of these drugs in Brazil compared to other countries. Therefore, information on the management of these patients, specifically concerning adverse events and the adjustment of ribavirin administration compared to other foreign services, may facilitate the safer handling of these drugs. However, the mortality rate observed in our cohort was similar to that observed in other real-life studies 22,28,29,32,33.

We believe that this study describes the largest cohort of patients infected with HCV genotype 1 and treated with BOC or TVR in combination with Peg-IFN and RBV in a real-life context in Latin America. We sought to include patients from different Brazilian service centers and with different characteristics to obtain a representative sample of patients during this phase of treatment for hepatitis C in Brazil. However, this study has some limitations. The first limitation is its cross-sectional, retrospective, observational, and multicentric nature. In this type of study, the leading center researchers involved are fully responsible for the choice of treatment offered and for providing the data. In this respect, the selection of patients for treatment and the choice between different treatments is at the discretion of the local investigator. This type of study may lead to selection bias for the patients included. Moreover, in this study, it was not possible to determine how many patients with chronic HCV infection and treatment indication were initially considered eligible for treatment or refused treatment. We cannot exclude the possibility that patients selected for treatment who had a higher likelihood of achieving SVR were included in our analysis. However, we believe that by following the standards described in the Brazilian protocol at the time 12,13 and including all patients treated consecutively in each center during the study period, we may have reduced the likelihood of selection bias.

Another potential bias in our study is the possibility of missing data, as only the data recorded in the medical records of the patients were included in the study. This has also been the case in other retrospective studies. In terms of monitoring of adherence to treatment, it was not possible to access this information due to the retrospective nature of this study. However, it is important to note that in our study, only variables that did not exceed 15% patient loss were included for analytical purposes. Conversely, to evaluate the effectiveness of treatment in clinical practice, real-life studies offer the advantage of eliminating the potential bias of a change in behavior and conduct that might occur during the execution of a prospective study 35. Therefore, the retrospective nature of our study may be advantageous because it provides a true assessment of the effectiveness of this type of therapy in Brazil.

According to data from the Brazilian Ministry of Health, approximately 7,800 treatments with BOC or TVR were performed at the national level between 2012 and 2016 36. We believe our sample represents a significant and representative percentage of this group of patients and provides valuable information about this experience with real-life data from treated patients.

In conclusion, although most of our patients achieved SVR after treatment, the rate of SAEs was very high in this population. Patients with cirrhosis had a lower risk of achieving SVR and an increased risk of developing SAEs. Therefore, despite the beneficial outcome and cure of a significant number of patients, the risk of the development of SAEs, including severe clinical complications and death, particularly in those with advanced liver disease, should be taken into consideration. In addition, the use of therapies such as BOC or TVR has been proscribed, and safer, more effective direct-acting drugs should be considered. Our results confirm the need to reformulate the guidelines for the treatment of chronic hepatitis C in Brazil, which recently incorporated safer and more effective drugs for the treatment of this condition.

## AUTHOR CONTRIBUTIONS

Callefi LA and Mendes-Correa MC were responsible for the study design, data collection and analysis and manuscript preparation. Villela-Nogueira CA contributed to data collection and participated in data analysis and manuscript revision. Tenore SB, Carnaúba-Júnior D, Coelho HS, Pinto PT, Nabuco LC, Pessoa MG, Ferraz ML, Ferreira PR, Martinelli AL, Chachá SG, Ferreira AS, Bisio AP, Brandão-Mello CE, Álvares-Da-Silva MR, Reuter T, Ivantes CA and Perez RM were responsible for data collection, participated in the data analysis and critically revised the manuscript. All authors read and approved the final version of the manuscript.

## Figures and Tables

**Table 1:** Baseline characteristics of patients with hepatitis C according to the antiviral treatment received.

VARIABLES	BOCEPREVIR (n = 158)		TELAPREVIR (n = 557)		*p*
	N	%	N	%	
Gender					0.246
Male	95	60.1	306	54.9	
Female	63	39.9	251	45.1	
Age (years)*					0.735
≤65	138	87.9	495	88.9	
>65	19	12.1	62	11.1	
BMI (kg/m^2^)**					0.341
Mean (SD)	27.4 (4.5)		27.3 (4.7)		
Median (min - max)	26.7 (20.6–41.6)		26.6 (18.0–43.4)		
Number of comorbidities					0.192
<2	108	68.4	410	73.6	
≥2	50	31.6	147	26.4	
Extrahepatic manifestations					<0.001
No	137	86.7	535	96.1	
Yes	21	13.3	22	3.9	
Previous hepatic decompensation					0.007
No	148	93.7	545	97.8	
Yes	10	6.3	12	2.2	
Genotype 1 subtype					0.314
1a	51	32.3	216	38.8	
1b	74	46.8	241	43.3	
1a/1b or 1	33	20.9	100	18.0	
Stage of liver fibrosis					0.096
F1+F2	18	11.4	35	6.3	
F3	51	32.3	189	33.9	
F4	89	56.3	333	59.8	
Treatment history					0.045
Relapser	45	28.5	141	25.3	
Non-responder	74	46.8	220	39.5	
Treatment-naïve	39	24.7	196	35.2	
Hemoglobin levels before treatment (g/dL)***					0.004
Normal	139	89.1	514	95.4	
Abnormal^a^	17	10.9	25	4.6	
Platelet count before treatment (per mm^3^)***					0.040
≥100,000	118	75.6	447	82.9	
<100,000	38	24.4	92	17.1	
Albumin levels before treatment (g/dL)^#^					0.760
≥3.5	147	96.1	510	95.5	
<3.5	6	3.9	24	4.5	
HCV RNA levels before treatment (IU/mL)^##^					0.756
<800,000	71	45.8	253	46.4	
≥800,000	84	54.2	292	53.6	

Missing data: (*) 1; (**) 110; (***) 20; (#) 28; (##) 15

a <12 g/dL (women), <13 g/dL (men)

BMI, body mass index; HCV, hepatitis C virus.

**Table 2:** Results of univariate and multivariate analyses of factors associated with sustained viral response in patients with hepatitis C infection.

	Univariate analysis			Multivariate analysis		
	PR	95% CI	*p*	PR	95% CI	*p*
Boceprevir (*versus* Telaprevir)	0.89	0.76–1.06	0.190			
Genotype 1 subtype			0.010			
1a	1					
1b	1.23	1.07–1.43				
1a/1b or 1	1.05	0.86–1.28				
Cirrhosis	0.66	0.59–0.75	<0.001	0.77	0.68–0.87	<0.001
Treatment history			<0.001			<0.001
Relapser	1			1		
Non-responder	0.58	0.49–0.68		0.69	0.59–0.81	
Treatment-naïve	0.79	0.69–0.91		0.83	0.73–0.95	
Abnormal hemoglobin levels before treatment*^a^	1.19	0.95–1.49	0.124			
Platelet count <100,000 per mm^3^ before treatment*	0.49	0.38–0.64	<0.001	0.6	0.46–0.77	<0.001
Albumin levels <3.5 g/dL before treatment**	0.76	0.50–1.15	0.193			
HCV RNA ≥800,000 IU/mL before treatment^#^	0.90	0.79–1.02	0.101			
RVR	1.98	1.64–2.40	<0.001	1.9	1.58–2.30	<0.001
Occurrence of SAEs	0.82	0.72–0.94	0.005			
Hepatic decompensation	0.56	0.36–0.88	0.012			
Infection	0.83	0.64–1.08	0.158			

Missing data: (*) 20; (**) 28; (#) 15

a <12 g/dL (women), <13 g/dL (men)

RVR, rapid viral response

PR, prevalence ratio

95% CI, 95% confidence interval.

**Table 3:** Distribution of patients with chronic hepatitis C according to the occurrence of SAEs and the treatment received.

VARIABLES	BOCEPREVIR (n=158)		TELAPREVIR (n=557)		*p*
	N	%	N	%	
Grade 3 or 4 anemia					0.042
No	128	81.0	407	73.1	
Yes	30	19.0	150	26.9	
Blood transfusion					0.014
No	148	93.7	482	86.5	
Yes	10	6.3	75	13.5	
Grade 3 or 4 rash					0.439^a^
No	155	98.1	538	96.6	
Yes	3	1.9	19	3.4	
Grade 4 neutropenia					0.222
No	144	91.1	523	93.9	
Yes	14	8.9	34	6.1	
Grade 3 or 4 thrombocytopenia					0.063
No	147	93.0	489	87.8	
Yes	11	7.0	68	12.2	
Hepatic decompensation					0.103
No	145	91.8	530	95.2	
Yes	13	8.2	27	4.8	
AE that led to discontinuation of treatment					0.587
No	129	81.6	465	83.5	
Yes	29	18.4	92	16.5	
Death					0.999^a^
No	157	99.4	552	99.1	
Yes	1	0.6	5	0.9	

AE, adverse event

a Fisher's exact test.

**Table 4:** Univariate and multivariate analysis of the occurrence of SAEs in patients with hepatitis C infection.

	Univariate analysis			Multivariate analysis		
	PR	95% CI	*p*	PR	95% CI	*p*
Female gender	1.47	1.25–1.73	<0.001	1.42	1.21–1.67	<0.001
Age >65 years*	1.50	1.24–1.82	<0.001	1.32	1.07–1.62	0.008
≥2 comorbidities	1.29	1.09–1.53	0.003			
Previous hepatic decompensation	1.46	1.05–2.03	0.023			
Cirrhosis	1.46	1.21–1.75	<0.001	1.25	1.04–1.52	0.019
Abnormal hemoglobin levels before treatment*^a^	1.48	1.16–1.89	0.001	1.56	1.23–1.98	<0.001
Platelet count <100,000 per mm^3^ before treatment*	1.72	1.47–2.02	<0.001	1.54	1.30–1.82	<0.001
Albumin levels <3.5 g/dL before treatment**	1.44	1.09–1.92	0.012			

Missing data: (*) 20; (**) 28

a <12 g/dL (women), <13 g/dL (men)

PR, prevalence ratio

95% CI, 95% confidence interval.

**Table 5:** Effectiveness and safety of real-life studies with boceprevir and telaprevir.

Author (Ref.)	PI	Country	n	Cirrhosis	SVR (%)	SAE (%)	Deaths (n)
Mauss (22)	BOC, TVR	Germany	1087	16%	58%	9%	3%
Backus (23)	BOC, TVR	USA	835	27% (BOC) 44% (TVR)	50% (BOC) 52% (TVR)	NA	NA
Sterling(24)/Gordon (33)	BOC, TVR	USA	2084	38%	44% (BOC) 54% (TVR)	12%	5
Calleja (28)	BOC	Spain	170	79%	47%	37%	2
Hezode (29)	BOC, TVR	France	511	100%	40%	50%	11
Callefi	BOC, TVR	Brazil	715	59%	56%	44%	6

Ref.: reference. PI: protease inhibitor. BOC: boceprevir. TVR: telaprevir. n: number. SRV: sustained viral response. SAE: serious adverse event. NA: not available.
